# Optimizing Radiation Dose Levels in Prospectively Electrocardiogram-Triggered Coronary Computed Tomography Angiography Using Iterative Reconstruction Techniques: A Phantom and Patient Study

**DOI:** 10.1371/journal.pone.0056295

**Published:** 2013-02-20

**Authors:** Yang Hou, Jiahe Zheng, Yuke Wang, Mei Yu, Mani Vembar, Qiyong Guo

**Affiliations:** 1 Department of Radiology, Shengjing Hospital of China Medical University, Shenyang, China; 2 CT Clinical Science Philips Healthcare, Cleveland, Ohio, United States of America; Virginia Tech, United States of America

## Abstract

**Aim:**

To investigate the potential of reducing the radiation dose in prospectively electrocardiogram-triggered coronary computed tomography angiography (CCTA) while maintaining diagnostic image quality using an iterative reconstruction technique (IRT).

**Methods and Materials:**

Prospectively-gated CCTA were first performed on a phantom using 256-slice multi-detector CT scanner at 120 kVp, with the tube output gradually reduced from 210 mAs (Group A) to 125, 105, 84, and 63 mAs (Group B–E). All scans were reconstructed using filtered back projection (FBP) algorithm and five IRT levels (L2-6), image quality (IQ) assessment was performed. Based on the IQ assessment, Group D(120 kVp, 84 mAs) reconstructed with L5 was found to provide IQ comparable to that of Group A with FBP. In the patient study, 21 patients underwent CCTA using 120 kV, 210 mAs with FBP reconstruction (Group 1) followed by 36 patients scanned with 120 kV, 84 mAs with IRT L5 (Group 2). Subjective and objective IQ and effective radiation dose were compared between two groups.

**Results:**

In the phantom scans, there were no significant differences in image noise, contrast-to-noise ratio (CNR) and modulation transfer function (MTF) curves between Group A and the 84 mAs, 63 mAs groups (Groups D and E). Group D (120 kV, 84 mAs and L5) provided an optimum balance, producing equivalent image quality to Group A, at the lowest possible radiation dose. In the patient study, there were no significant difference in image noise, signal-to-noise ratio (SNR) and CNR between Group 1 and Group 2 (p = 0.71, 0.31, 0.5, respectively). The effective radiation dose in Group 2 was 1.21±0.14 mSv compared to 3.20±0.58 mSv (Group 1), reflecting dose savings of 62.5% (p<0.05).

**Conclusion:**

iterative reconstruction technique used in prospectively ECG-triggered 256-slice coronary CTA can provide radiation dose reductions of up to 62.5% with acceptable image quality.

## Introduction

In the past decade, with the development of multi-detector spiral computed tomography (CT), coronary computed tomography angiography (coronary CTA) has increasingly become a noninvasive method of choice for the rule-out of coronary artery disease [1]–[5] owing to its high sensitivity. However, despite radiation dose reduction technologies implemented by the CT manufacturers, concerns persist. Therefore, coronary CT should continue to follow the principle of “ALARA” (as low as reasonably achievable).

The use of various basic techniques for coronary CTA such as decreasing tube voltage and current, shortening/optimizing scan length, electrocardiogram (ECG) tube current modulation, prospective gating and application of prospectively ECG-triggered, high-pitch scan mode have resulted in halving radiation dose every two years since 2005 [6]. However, in order to consistently achieve further radiation dose reductions, these methods have to be supplemented by newer reconstruction technologies which can help address the limitations (i.e., increased image noise) of the currently used filtered back projection (FBP) reconstruction algorithms. Iterative reconstruction techniques (IRT) have been recently introduced, offering an innovative solution for reducing CT radiation dose. They can markedly lower image noise, effectively increase signal-to-noise ratio (SNR) and contrast-to-noise ratio (CNR), and achieve equivalent, or in some cases, better image quality (IQ) compared to routine-dose FBP reconstruction. Recent work has shown that IRT can enable dose reductions in chest and abdominal CT in the range of 32–65% depending on the patients’ body mass index (BMI) [7]–[15]. Likewise, the use of IRT has also been investigated in coronary CTA, demonstrating radiation dose reductions in the range of 40–76% compared to FBP while retaining the image quality [7]–[21].

However, these early works did not investigate the level of tube output reductions (along with the corresponding levels/strengths of IRT) that can be safely employed beyond which the image quality will be negatively impacted. For this reason, we first performed prospectively ECG-triggered 256-slice multi-detector coronary CTA in phantoms, with a constant tube voltage (120 kV) and a ‘standard’ tube current, followed by scans performed with gradually decreasing tube currents. Images from scans obtained with decreasing tube output were reconstructed with corresponding levels of IRT to maintain a certain noise level and compared to FBP reconstructions used for the scans performed with the standard tube output. Once a threshold of tube output reduction was identified (with the corresponding IRT level) that would provide IQ comparable to FBP from the standard scans, this was extended to a patient study, with prospectively gated axial coronary CTA performed in two demographically matched cohorts – one scanned with standard tube output and reconstructed with FBP and the other with tube reduction determined from the phantom scans and reconstructed with the appropriate level of IRT. Thus, the minimal radiation dose for 256-slice multi-detector coronary CTA using IRT without loss of clinical information could be determined.

## Materials and Methods

### Ethics Statement

The study protocol was approved and the study was performed under the supervision of the ethics committee of the Shengjing Hospital of China Medical University (Shenyang 110004, China) (no. 2012PS26K). Participants provided their written informed consent to participate prior to the study onset.

### Phantom Study

Catphan 500 phantom (Phantom Laboratory, Cambridge, NY, USA) with CTP528 and CTP401 modules were used to evaluate objective image quality (image noise, CNR and modulation transfer function (MTF)) and subjective image quality (artifact, nodule conspicuity, detectable minimal nodule size, overall image quality).

#### Acquisition protocol

We used prospective ECG-gated scanning and image reconstruction on a 256-slice multi-detector CT scanner (Brilliance iCT; Philips Healthcare, Cleveland, OH, USA). The scan parameters include a detector configuration of 128×0.625 mm (detector collimation); slice thickness 0.9 mm; gantry rotation time 0.27 second; display field-of-view, 22 cm; and scan length 8 cm. An ECG signal was generated using a simulator set to a heart rate of 60 beats/min. Scans were performed using our institutional protocol (i.e., routine protocol), with the tube voltage set to 120 kVp and the tube current – x-ray ON time product (i.e., the tube output) of 210 mAs (i.e., Group A). The trigger time was set at 75% of the R-R interval without the buffer zone. With the tube voltage kept at 120 kVp, the tube output was gradually reduced to 125 mAs (Group B), 105 mAs (Group C), 84 mAs (Group D), and 63 mAs (Group E). The reduction rate in radiation dose for each effective tube current time product was 40%, 50%, 60%, 70%, compared to our routine dose.

#### CT image reconstruction and evaluation

The routine-dose and reduced-dose scan series were reconstructed using FBP and IRT (iDose^4^, Philips Healthcare, Cleveland, OH, USA). The description of the IRT used in this study has been explained in prior work [17]. Five IRT levels (L2, 3, 4, 5, 6) were used in each scan series with a coronary kernel (XCC). These levels were designed to attain a certain noise reduction factor to compensate for any noise increase resulting from lowering the tube output. [16], [17] Thirty scans were reconstructed to evaluate the noise and contrast to noise ratio (CNR).

#### Objective image quality: Measurement of image noise, CNR and MTF

For each CT images series, image noise and CNR were measured by a radiologist blinded to the scanning and reconstruction methods at the CTP401 module of the Catphan phantom; the noise was defined as the standard deviation (SD) of pixel values in background within a 10-mm diameter circular region of interest (ROI). The CT number of the Teflon objects in a 13-mm diameter area in the same slice was measured using a circular ROI cursor. CNR values were calculated as follows: (CTa-CTb)/SDb, where CTa and CTb are the CT numbers of the acrylic objects and the background ROI, and SDb is the standard deviation of the attenuation values of the background [20]. Mean noise and CNR were calculated from the noise and CNR measurements of 10 continuous images in Z direction.

**Figure 1:**
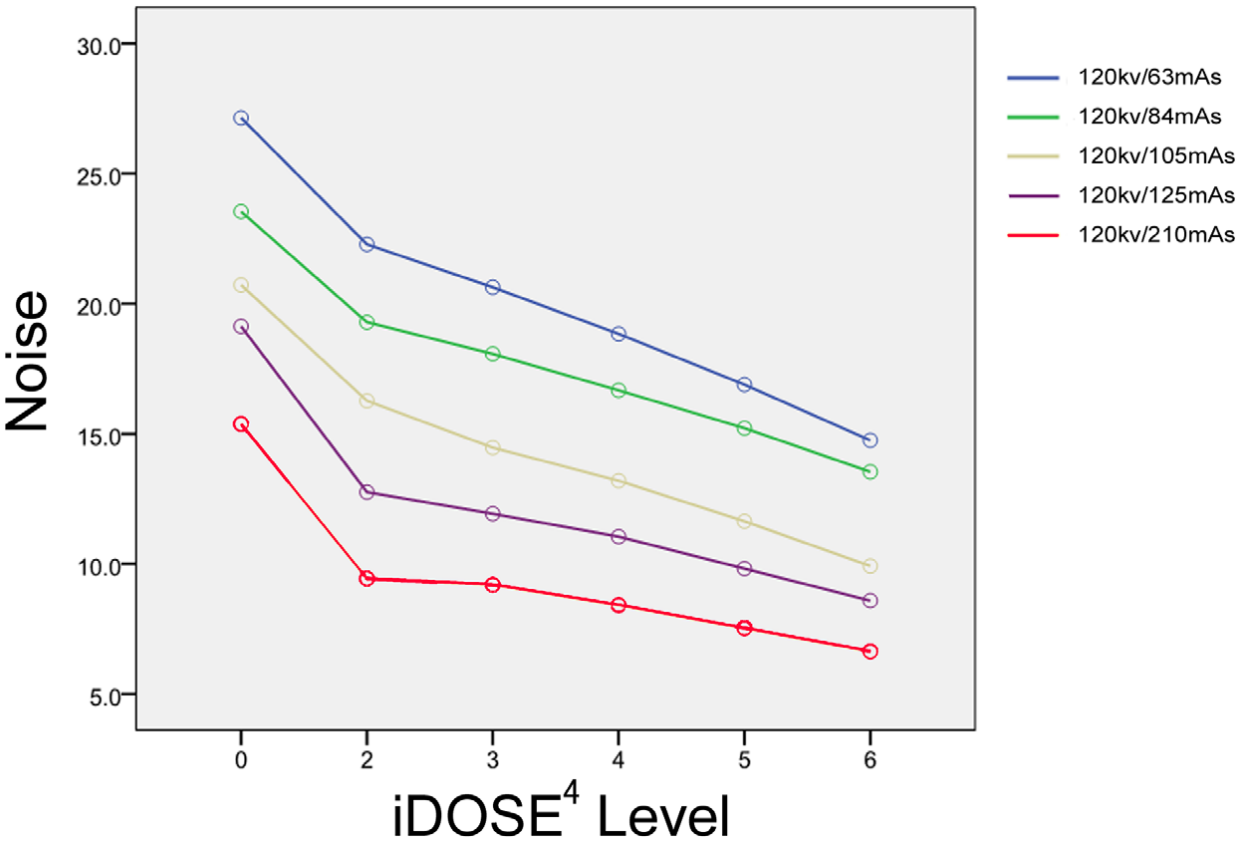
Noise reduction in images reconstructed with iDOSE^4^ in phantom. At equal radiation doses, iDose^4^ reconstruction algorithm yielded lower image noise than FBP reconstruction algorithm, and with increased iDose^4^ level, image noise decreased in a linear manner.

**Figure 2:**
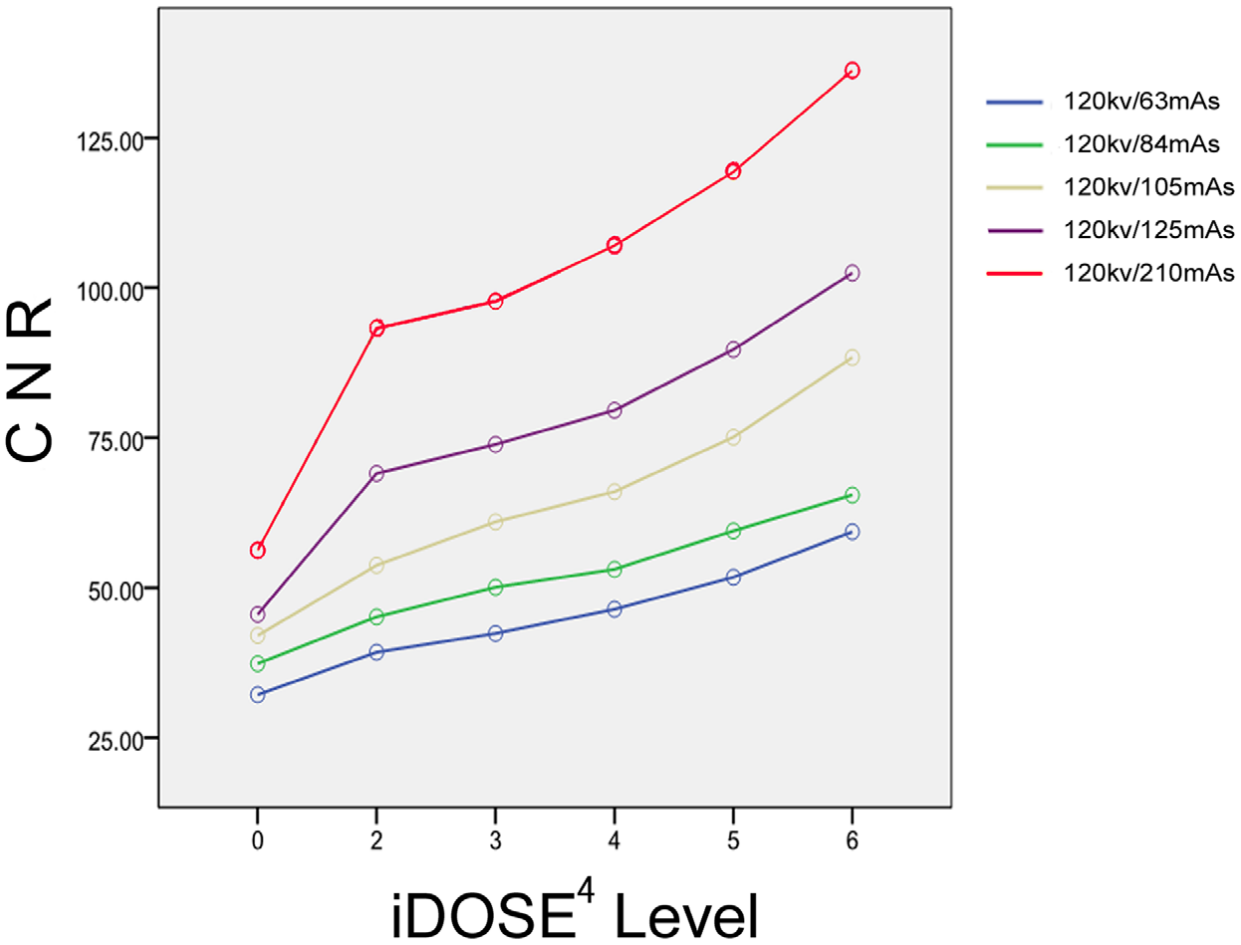
CNR increase in images reconstructed with iDOSE^4^ in phantom. At equal radiation doses, iDose^4^ reconstruction produced higher CNR than FBP reconstruction, and the CNR was increased in a linear manner with increased iDose^4^ level.

The effects of different radiation doses and different IRT levels on image noise and CNR were investigated. noise and CNR curves are shown in Figures 1 and 2. The IRT images from each low-dose group with a noise value similar to images reconstructed by routine-dose FBP reconstruction algorithm were included for later analysis.

The modulation transfer function (MTF) was measured from CTP 528 module images reconstructed using FBP from routine-dose scans (120 kV, 210 mAs) and IRT from the reduced dose scans and the curves were plotted.

#### Evaluation of subjective image quality

The subjective image quality (IQ) scoring was performed on CTP 401 module images reconstructed using FBP on routine-dose (120 kV, 210 mAs) scans (Group A) and and IRT from reduced-dose scans (Groups B:125 mAs, C:105 mAs, D:84 mAs, E:63 mAs). The artifacts (including graininess, streaks, plastic-look), nodule conspicuity, detectable minimal nodule size, and overall IQ of images were evaluated. The above mentioned images were independently scored by two experienced radiologists blinded to the reconstruction algorithm. The objective IQ of IRT reconstructions was recorded on a 4-point scale, where 1 =  unacceptable with respect to diagnostic quality, 2 =  suboptimal diagnostic quality and poor image quality compared to Group A, 3 =  good diagnostic quality and image quality equal to Group A, and 4 =  excellent diagnostic quality and superior image quality compared to Group A. With respect to minimal detectable nodule size, the acrylic spheres we used ranged from 2 to 10 mm. Data were recorded. The mean scores from these two radiologists were used as the final scores. Based on above subjective and objective indices, the IRT reconstructions with IQ equivalent to routine-dose reconstruction (Group A) were selected.

### Patient Study

#### Population studied

Over a period of two months (March-April 2012), a total of 56 consecutive patients (34 males and 23 females) were prospectively enrolled in the study. In the first month, 21 patients underwent coronary CTA using the routine institutional protocol (120 kV, 210 mAs) (Group 1). In the second month, 36 patients underwent coronary CTA using a reduced tube-output (120 kV, 84 mAs) (Group 2). The study protocol was approved by our institution’s ethics committee, and written informed consent was obtained from each subject.

Exclusion criteria: BMI<20 or>30, severe renal inadequacy (creatinine clearance rate ≤120 µmol/L); pregnant; known allergies to iodinated contrast agent; severe arrhythmia; cardiac function or thrombolysis in myocardial infarction (TIMI) flow<Grade III after coronary artery stenting or coronary artery bypass grafting (CABG).

#### Acquisition protocol

A 256-slice MDCT scanner (Brilliance iCT; Philips Healthcare, Cleveland, OH, USA) was used. Automatic bolus tracking (Bolus Pro, Philips Healthcare) was used with a region of interest (ROI) in the ascending aorta at the level of pulmonary artery. The scans were initiated under full inspiration 6 seconds after a pre-determined signal attenuation threshold of 180 HU was attained. A volume of 60–70 mL of contrast media (Iohexol 350; GE Healthcare, Shanghai, China), followed by 20 mL saline was intravenously injected at a flow rate of 5 mL/s into the antecubital vein through the use of dual-tube high pressure syringe (Ulrich REF XD 2051) equipped with an 18-gauge catheter. The coronary CTA scan parameters were as follows: tube potential = 120 kVp; effective tube current-time product = 210, 84 mAs for routine-dose group and low-dose group, respectively; detector configuration = 128×0.625 mm; rotation time = 270 ms; field of view = 250 mm; slice thickness = 0.9 mm; increment = 0.45 mm. The scan trigger was centered around a physiologic cardiac phase of ventricular diastasis corresponding to 75% of the R-R interval, with a ±5% buffer used when the fluctuation of heart rate was >5 beats/min. Prior to CT examination, patients with heart rate >70 beats/min were administered oral ß-receptor blockers 12.5–25 mg (Metoprolol Succinate sustained-release tablets, AstraZeneca, Sweden) to decrease and stabilize the heart rate.

#### CT data reconstruction and image analysis

Images from the routine-dose (Group 1) acquisitions were reconstructed using FBP algorithm. The low-dose acquisitions (Group 2) were reconstructed using an IRT with the appropriate level (L5) to account for any increase in noise caused by the lowered tube output. A standard-sharp reconstruction kernel (XCC) was used in all image reconstructions. The matrix size of reconstructed images was 512×512.

#### Subjective evaluation of image quality

Transverse image data from each group was analyzed using an advanced cardiac application (Cardiac Viewer and Comprehensive Cardiac Analysis) on a dedicated CT workstation (Extended Brilliance Workspace Version 4.01, Philips Healthcare). In addition to the transverse images, curved multi-planar reformats of the left anterior descending (LAD) artery, left circumflex (LCX) artery, and right coronary artery (RCA) were used to evaluate image quality as judged by overall image acceptability, contrast between blood vessels and the surrounding tissues, lumen edge sharpness and subjective noise in vessels with diameters ≥1.5 mm. These characteristics were evaluated using a 4-point grading scale: 4: excellent image quality, very good contrast, very clear and sharp lumen edge, and very low noise; 3: good image quality, good contrast, sharp lumen edge, and low noise; 2: fair image quality and contrast, blurred lumen edge, and high noise; 1: poor image quality and contrast, blurred lumen edge, and very high noise. Image quality was evaluated by two experienced (>5 years) cardiac radiologists who were blinded to scan conditions and patients’ clinical data. If necessary, a third radiologist was asked to adjudicate the differences in order to obtain a consensus score.

#### Objective evaluation of image quality

A 2-cm^2^ROI was identified within the aortic root at the level of the origin of the left main coronary artery. The mean CT value (HU) within the ROI was measured and its standard deviation was noted down as the image noise of the aorta. The SNR was calculated using the formula: SNR = signal_ao_/noise_ao_. The CT value of pericardial fat surrounding the left main coronary artery at the same level was measured, and the ROI size was designated as the maximum area without vessels. The CNR was calculated using the formula: CNR = (HU_ao_ – HU_fat_)/image noise_ao_. [22].

#### Measurement of radiation dose

The CTDI_vol_ and dose-length product (DLP) values during the scans were recorded. The estimated effective dose (ED) was derived as the product of the DLP and a conversion coefficient *k*, (ED = DLP×*k*), where *k* is the conversion coefficient for chest (*k* = 0.014 mSv mGy^−1^ cm^−1^) [23].

### Statistical Analysis

Statistical analysis was performed using commercially available software (SPSS 17.0, SPSS, Chicago, IL, USA). Continuous variables were expressed as mean ± standard deviation. Continuous variables and demographic data were compared by a paired Student’s *t*-test. The kappa test was used to test inter-reader agreement in subjective evaluation of image quality. When discrepancy exists, a third radiologist was asked to give evaluation, and the consistent score was taken as the final scoring result. If the score from the three radiologists was different, the radiologists would discuss the case until an agreement was reached. The Mann-Whitney U test was used to test for differences in subjective evaluation of image quality between two groups. A level of P<0.05 was considered statistically significant.

## Results

### Phantom Study

#### Comparison of phantom objective indices

As expected, a reduction in the tube output resulted in corresponding increases in the image noise. The use of the IRT addressed this increase in image noise. For a given tube output, the IRT reconstruction algorithm yielded lower image noise compared to FBP, with increasing levels of IRT offering increasing noise reductions in a linear manner (Figure 1).

There were no significant differences in mean CT values of the background and the Teflon spheres between each low-dose group and routine-dose FBP group (*p* = 0.4). At equal radiation doses, IRT reconstruction produced higher CNR than FBP reconstruction, with the CNR increasing in a linear manner with increased IRT level (Figure 2).

IRT levels L3, L4, L5, L6 were used for 125 mAs (Group B), 105 mAs (Group C, 84 mAs (Group D), and 63 mAs (Group E) sequences respectively, to compensate for the increased image noise caused by decreased tube output to achieve the noise and CNR equivalent to or better than those of images reconstructed with FBP at 120 kV and 210 mAs.

There were no significant differences in image noise and CNR between routine-dose FBP group (Group A) and the 84 mAs, 63 mAs groups (i.e., Groups D and E respectively). The image noise and CNR in the Groups B and C were significantly superior to those of the routine-dose FBP group (Group A). The image noise and CNR in each group are shown in Table 1.

**Table 1:** Image noise and CNR in each phantom group.

Item	120 KV+210 mAs/FBP (Group A)	120 KV+125 mAs/L3 (Group B)	120 KV+105 mAs/L4 (Group C)	120 KV+84 mAs/L5 (Group D)	120 KV+63 mAs/L6 (Group E)	F	P
Image noise	15.4±0.8	11.9±1.7	13.2±0.6	15.2±1.1^†^	14.8±1.4^†^	16.4	<0.001
	(14.1–16.9)	(10.6–16.2)	(12.2–14.1)	(13.5–16.7)	(13.0–17.8)		
CT value of Teflon spheres	965.8±3.7	968.2±2.0	967.8±2.8	966.1±6.4	965.3±3.4	1.1	0.4
	(962.0–973.9)	(965.2–971.7)	(963.9–973.3)	(958.1–975.0)	(957.7–970))		
CT value of background	100.9±8.4	98.7±1.3	97.7±1.4	98.0±1.8	98.7±2.0	0.9	0.4
	(97.3–124.6)	(96.0–99.9)	(95.5–100.2)	(95.1–101.6)	(96.4–102.5)		
CNR	56.2±2.8	73.9±8.4	66.0±3.0	59.5±2.3^†^	59.3±5.3^†^	20.7	<0.001
	(51.3–61.0)	(53.9–82.0)	(53.9–82.0)	(56.9–63.8)	(48.7–66.8)		

Note: ^†^indicates no significant statistical difference compared to group A; CNR: contrast-to-noise ratio; L: IRT level.

**Figure 3:**
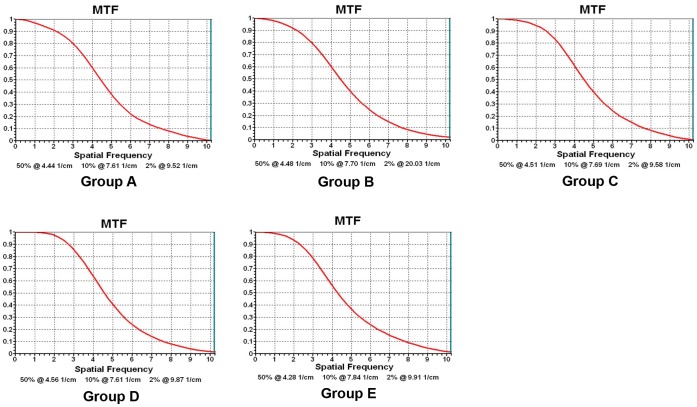
MTF curves of the phantom scans. No significant differences are observed between Group A and each of the low-dose iterative reconstruction groups (Groups B–E).

There was no significant difference in MTF curves between Group A and low-dose iterative reconstruction groups (Groups B–E), *i.e*., there was no significant difference in spatial resolution among the groups (Figure 3).

#### Comparison of phantom subjective image quality

**Figure 4:**
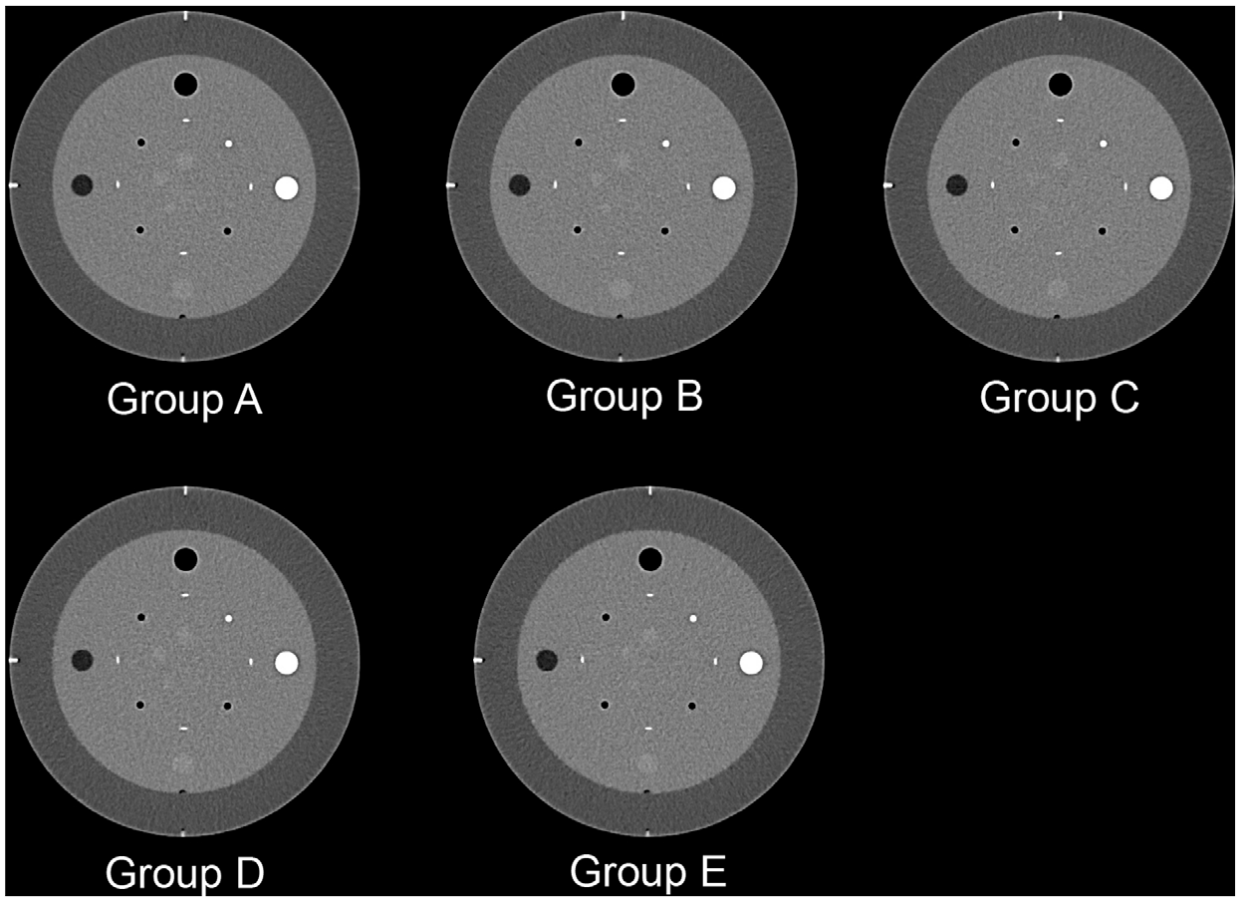
Phantom images acquired with routine dose(120 kv, 210 mAs) and different low dose (120 kv, 125–63 mAs) scan. A: 120 KV, 210 mAs with FBP reconstruction. B: 120 KV, 125 mAs with iDose^4^ reconstruction (L3). C: 120 KV, 105 mAs with iDose^4^ reconstruction (L4). D: 120 KV, 84 mAs with iDose^4^ reconstruction (L5). E: 120 KV, 63 mAs with iDose^4^ reconstruction (L6). The subjective image quality score was equivalent between Group D and Group A. The score of nodule conspicuity in the Group E was significantly lower than that in the Group A.

The center-slice images of CTP401 module in each dose group are shown in Figure 4. The subjective image quality scores in Groups B and C were better than those of Group A. The subjective image quality score was equivalent between Group D and Group A. The score of nodule conspicuity in the Group E was significantly lower than that of Group A, and the detectable minimal nodule size in Group E reached 6 mm, which was higher compared to the other groups (Table 2).

**Table 2:** Subjective image quality scores for phantoms in different dose groups.

Item	120 KV+210 mAs/FBP (Group A)	120 KV+125 mAs/L3 (Group B)	120 KV+105 mAs/L4 (Group C)	120 KV+84 mAs/L5 (Group D)	120 KV+63 mAs/L6 (Group E)
Artifact	3	4	3	3	3
Nodule conspicuity	3	3	3	3	2
Detectable minimal nodule size (mm)	4	4	4	4	6
Overall image quality	3	4	3.5	3	2

FBP: filtered back projection; L: IRT level.

Taken together, Group D (120 kV, 84 mAs and L5 reconstruction) provided an optimum balance, producing equivalent image quality to Group A, at the lowest possible radiation dose.

### Patient Study

All subjects underwent coronary CTA successfully. There were no significant differences in general data of subjects between Group 1 and Group 2 (Table 3).

**Table 3:** Comparison of general data and objective image quality of patients between Group 1 (routine-dose FBP reconstruction) and Group 2 (120 KV+84 mAs/IRT L5 reconstruction).

Item	Group 1	Group 2	t	*P*
Age (year)	54±18 (34–75)	52±10 (28–77)	−0.58	0.56
Sex (male/female )	15/6	20/16	2.97^†^	0.09^†^
Body mass index	25.40±2.06	25.45±2.15	−1.28	0.2
	(22.83–28.68)	(23.16–29.10)		
Heart rate	62±6 (47–69)	59±6 (49–69)	−1.16	0.25
Heart rate viability	1.72±1.15 (0–5)	1.16±1.07 (0–5)	−1.88	0.07
Scan length	12.92±1.62	12.76±1.20	−0.44	0.66
	(10.92–16.35)	(10.96–18.78)		
CT_aorta_	421.30±43.63	444.46±50.85	−1.75	0.09
	(354.4–558.1)	(352.7–540.1)		
CT_fat_	−99.21±18.84	−91.97±17.92	1.44	0.16
	(−74.5–−151.3)	(−59.0–−124.3)		
Image noise	35.49±9.44	36.54±10.70	0.37	0.71
	(24.0–59.1)	(19.1–69.4)		
SNR	12.43±2.54	13.63±4.93	1.03	0.31
	(7.1–16.5)	(5.8–30.7)		
CNR	15.39±3.22	16.28±5.41	0.68	0.50
	(8.7–20.1)	(7.0–35.1)		

Note: ^†^indicates the results of *x* ^2^. SNR: Signal-to-noise ratio, CNR: contrast-to-noise ratio.

There were no significant difference in image noise, SNR and CNR between Group 1 and Group 2. The subjective indices in Group 2 were slightly better than those in the Group 1 (Table 3).

**Figure 5:**
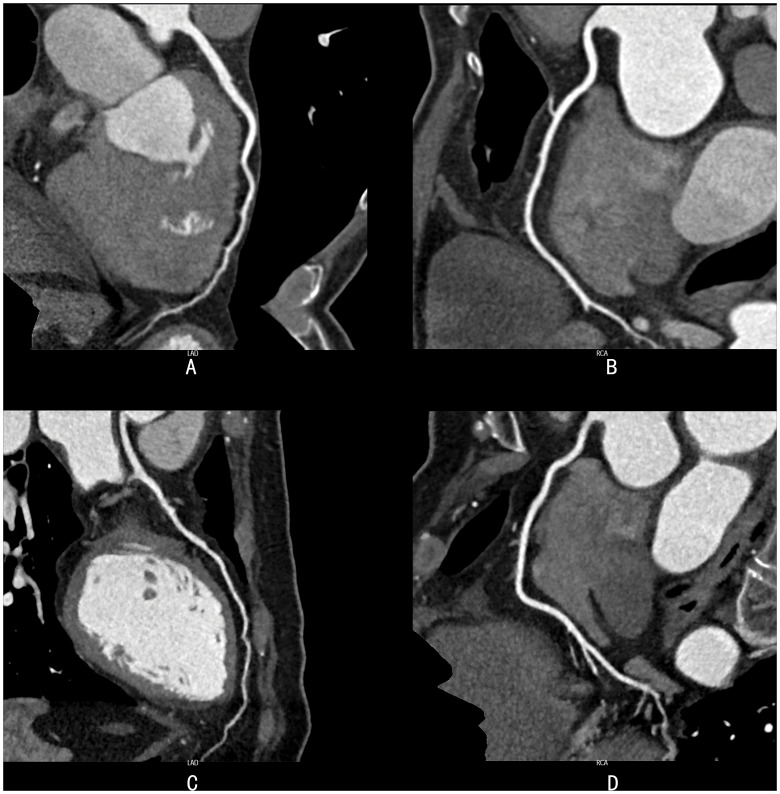
Representative examples of patient scans. There were no significant differences in subjective ranking for image sharpness, contrast, noise, and overall acceptability between patients belonging to Group 1 and 2. (A, B): Group 1, CPR pictures of LAD (Fig. 5A) and RCA (Fig. 5B) of a 53-year-old male with a body mass index (BMI) of 24.2 scanned at 120 kVp and 210 mAs and reconstructed with filtered back projection. Image quality (IQ) scores were 4, 4, 4, and 4 for contrast, sharpness, subjective noise, and acceptability, respectively. (C, D): Group 2, A 57-year-old male with BMI of 24.1 scanned at 120 kVp (Fig. 5C) and 84 mAs (Fig. 5D) and reconstructed with L5. IQ scores were 4, 4, 4 and 4 for contrast, sharpness, subjective noise, and acceptability, respectively.

The two radiologists showed very good consistency in subjective scores. The kappa value of image contrast, sharpness, objective noise and overall image quality was 0.66, 0.84, 0.84, and 0.85, respectively. In both groups, each subjective index was scored ≥3, and the IQ was good and met clinical diagnostic requirement. There were no significant differences in the index scores between the groups (Table 4, Figure 5).

**Table 4:** Comparison of subjective image quality between Group 1 (routine-dose FBP reconstruction) and Group 2 (120 KV+84 mAs/IRT L 5).

Item	Group 1(score 4/3/2/1)	Group 2(score 4/3/2/1)	U	*P*
Contrast	20/1/0/0	35/1/0/0	370.5†	0.69
Sharpness	20/1/0/0	31/5/0/0	343.5†	0.28
Objectivenoise	19/2/0/0	32/4/0/0	372†	0.85
Overall Imagequality	19/2/0/0	30/6/0/0	351†	0.46

† Mann-Whitney test were used.

The CTDI and DLP were 19.55±4.11 (Group 1) and 228.55±41.14 (Group 2), showing significant differences (p<0.001). The effective radiation dose in Group 2 was 1.21±0.14 mSv compared to 3.20±0.58 mSv (Group 1), reflecting dose savings of 62.5% (p<0.05).

## Discussion

In our study, we scanned the Catphan CT phantoms using a conventional tube voltage and decreasing tube currents, measured noise and CNR of each dose group reconstructed using both FBP and IRT approaches, and identified the levels of IRT in each low dose group that provided IQ that was equivalent to the routine-dose FBP reconstruction. From the comparisons of IQ (both subjective and objective) between the routine-dose FBP group and each low- dose IRT group, the protocol of 120 kV, 84 mAs and L5 reconstruction was determined as the protocol that provided an optimal balance of IQ and radiation dose reductions. This protocol was also performed in patients with normal body habitus prospectively enrolled in a clinical study to further confirm the feasibility of this low-dose protocol.

The IRT used in this study (iDose^4^) is a fourth generation algorithm designed to reduce image artifacts and noise while maintaining the structural/anatomical information [16], [17]. The use of photon statistics in the projection domain helps to iteratively reduce noise and preserve edges, and use of noise/structure models in the image domain to further reduce image noise.

The levels of IRT used in this study are designed to provide a noise reduction factor to compensate for any noise increases with the reduction in the tube output. Our phantom results showed a linear decrease in image noise with increasing levels of IRT; our experience was similar to prior work [16], [17] achieving noise reduction levels of 15%–45% as the levels of the IRT was increased from L2 to L6 for a given tube output. Thus, when the tube output is reduced by 40–70%, the corresponding IRT levels can be employed to result in objective image quality which is equivalent to or better than the FBP images from the routine-dose scans (i.e., Group A). In our phantom study, the noise values in the Groups B (125 mAs) and C (105 mAs) were actually lower than that those measured in Group A, and there were no significant differences in actual noise values between Groups D & E and Group A. Lowered image noise resulted in increased CNR in Groups B and C compared to Group A; no significant differences in CNR were observed between Groups D & E (59.3±5.3 and 59.5±2.3 respectively) and Group A (56.2±2.8)(*P*<0.001).

There were no significant differences in MTF value and curve shape between Group A and Groups B–E. This suggests that the IRT decreases image noise, while not negatively impacting spatial resolution. Retaining a relatively high spatial resolution is of importance for displaying coronary arteries.

The subjective evaluation scores of phantoms were similar between each low-dose iterative reconstruction group (Groups B–E) and Group A. No graininess, streak artifacts or blotchy/plastic artifacts were observed in each group. This advantage is superior to early iterative reconstruction approaches [24]. This can be attributed to the IRT used in this study which reduces noise without altering the image noise power spectrum and prevents artifacts to preserve the natural appearance of the image.

The subjective scores in Groups B and C were superior to those of Group A. Artifact, nodule conspicuity, minimal detectable nodule size and overall IQ in Group D were equivalent to those in the Group A. The nodule conspicuity and minimal detectable nodule size in the Group E were slightly lower than those in the Group A. These results may be related to the slightly decreased spatial resolution [11]. Our phantom experimental results showed that according to the “ALARA” principle, a tube voltage of 120 kVp, 84 mAs tube current x-ray on time product supplemented by an iterative reconstruction technique of an appropriate level (L5) is the optimal combination that provides IQ comparable to the FBP reconstructions of routine-dose scans.

Recently, some studies investigating low radiation dose coronary CTA used a decreased tube voltage (80 kVp and 100 kVp) and maintained high IQ and CNR in a select group of patients with a normal body mass index [25]–[30]. This could be attributed to the selected energy levels being closer to the K edge of iodine, thereby increasing the contrast enhancement and thus potentially enabling a reduction of volume of contrast agent used.

However, the use of low tube voltage could also result in a larger proportion of dose absorbed by the body, potentially causing a higher degree of beam hardening effects. Since reducing the tube output (current) instead of the tube voltage only changes the effective energy and affects the x-ray penetration to a lesser extent, we used a conventional tube voltage (120 kVp) and low tube currents in this study.

The prospectively ECG-triggered coronary CTA is currently the main method to decrease the radiation dose in coronary CTA. The 256-slice multi-detector computed tomography system can achieve an axial scan range of 8 cm and allow scan of the entire heart within 3 cardiac cycles, with a rotation speed of 0.27 sec, which could relax the heart rate requirements for coronary CTA using prospective gating (i.e., increases the threshold of the upper HR limit to 75 bpm) while at the same time enabling coronary CTA imaging at well under the average background radiation levels [22], [31], [32].

Extending our findings from the phantom scans, we have shown that the iterative reconstruction technique used in this study (iDose4), combined with prospective ECG gating in 256-slice MDCT can significantly decrease radiation dose in patient scans without negatively impacting IQ. Results from this study showed that there were no significant differences in subjective and objective IQ between Group 2 (reduced dose patient scans) and Group 1 (routine dose). The CTDI and ED in the Group 2 were decreased by 63% and 62% respectively compared to Group 1 (p<0.05). The effective radiation dose in the Group 2 reached 1.21±0.14 mSv. The extent of radiation dose savings is similar to recent findings [16], [19]. The dual benefits of maintaining (or even improving IQ) at low radiation dose could make the use of IRT in virtually all patient cohorts, especially in those sub-groups that are sensitive to radiation dose (younger population like infants and pediatrics) and those who require re-examination for follow-up of coronary plaque progression, percutaneous transcoronary angioplasty, coronary artery bypass grafting (CABG), etc.

The limitations of this study are as follows: (1) Our sample size was small and we did not adjust the protocol according to the BMI/body weight of the individual patients (the BMI ranged from 23 to 29 in our study). (2) We only focused on the overall image quality and did not investigate the effect of the IRT on coronary artery plaque and the diagnostic accuracy by comparing with the respective gold standards. (3) We did not evaluate the reconstruction time of the IRT compared to the conventional FBP, which may influence its wide clinical application; but the IRT used in this study had a reconstruction time of about 20 images per second making it clinically practical.

### Conclusion

In conclusion, our results of phantoms and the subjects with normal body weights confirmed that the iterative reconstruction technique (iDose^4^) used in prospectively ECG-triggered 256-slice coronary CTA can greatly decrease image noise and improve image quality, while at the same time providing radiation dose reductions of up to 63%.
